# Special Issue: Plant Metabolomics

**DOI:** 10.3390/metabo10110467

**Published:** 2020-11-16

**Authors:** Sándor Gonda

**Affiliations:** Department of Botany, Division of Pharmacognosy, University of Debrecen, Egyetem tér 1, 4032 Debrecen, Hungary; gonda.sandor@science.unideb.hu

This Special Issue was initiated to collect a handful of studies on plant chemistry, utilizing metabolomics as the main technique, to show the diversity of possible applications of this approach. Metabolomics aims to characterize to capture a snapshot of the whole metabolome, which is defined as the complete small-molecule pool in a biological matrix [1]—this not only includes specialized (secondary) metabolites, but also metabolic intermediates, hormones, and so on. The term “metabolome” was coined as an analogue to the terms “proteome” and “transcriptome” which refer to the whole set of proteins and gene transcripts in a biological sample, respectively.

“Omics” sciences all aim to capture a snapshot of the whole variability of a biological domain, for example transcriptomics aims to characterize the whole transcriptome. There is an unbelievable diversity of features at any level of biological systems: even at the level of individual organisms, the variability of the genome, the transcriptome, proteome and the metabolome can be considered, let alone complex interactions between species, and ecosystem-level networks of competition and cooperation, mediated by various proteins and metabolites [2].

Plant metabolites have been used for millennia for pharmaceutical and technological (e.g., preservation, dyes) as well as nutritional purposes. With technological development, it was made possible to characterize the chemical compounds behind the bioactivities, resulting in the development of natural product research, chemo-taxonomy, as well as many other disciplines. In recent years, new technologies in chemical analysis became available to a wider community of researchers, which resulted in a previously unimaginable depth of chemical characterization possibilities.

Metabolomics enables partial chemical characterization of biological and other samples by putative annotation of the compounds within them, without the need to use authentic standards. Compounds in a biological matrix vary in their chemical character; what is more, their concentration range spans several orders of magnitude [3]. Therefore, it is no surprise that no single instrumental method can cover all metabolites in a single analysis. Still, the amount of information gathered about a sample is much higher than in the case of traditional approaches, because of the high coverage and relatively high throughput: a few hundreds to a few thousands of features are usually detected in an experiment.

The above-mentioned coverage and diversity is well exemplified by some articles in this Special Issue [4,5,6]. Hence, metabolomics has become a widely applied means of chemical characterization. The current Special Issue is a great example of the potential of the technique: phenomena studied in the articles of the Special Issue include plant-microbe interactions [5,6,7], phenological development [8], pollination [9], post-harvest technological issues [10,11], comparison of species for bioactive compound patterns [4,12], as well as other approaches.

In-depth analysis of such chemical diversity is now made possible by metabolomic techniques on various instrumental platforms. Though metabolomic studies can also be run on NMR [11] and GC-MS [7], the most widespread technique is LC-MS (LC-ESI-MS/MS). This is in part because of the highest metabolome coverage, as well as the flexibility of sample preparation—many compound classes can be focused on with the technique. Of course, maximizing the coverage of the set of natural products is not that straightforward, as shown by a recent study on *Asparagus* [13].

The only way to obtain information on absolute concentration, and unequivocal identification (i.e., distinguishment of isomers) is the usage of authentic standards. A few studies have used this approach, including, a study on *Trollius* polyphenolic substances [12], and an excellent study utilizing a wide range of iridoid glycosides, xanthones and flavonoids and as authentic standards [4]. In a headspace GC-MS study of endophytic fungi-derived volatile organic compounds (VOCs) [7], identification of compounds is done in an unambiguous manner with authentic standards; though in this case, quantitation is not that straightforward as in liquid injection techniques.

Specialized metabolites have always been important compounds in phytochemistry, and several studies were centered around hypotheses in this field: flavonoid glycosides and tannic-like compounds from *Alnus* species [14], and tomato priming by growth promoting rhizobacteria [5].

Other studies used metabolomic analysis of mainly primary metabolites (intermediates of core metabolism: amino acids, sugars, etc.) followed by pathway analysis to investigate various problems in plant physiology. This set includes a publication examining tomato fruit metabolites during Mg oversupply [15] and a paper on primary metabolism of mutant *Arabidopsis* lines during ABA treatment [16]. Excellent studies on primary metabolism also include one on partially submerged stress in deepwater rice [17], and a study on the effects of phenological development on the leaf metabolome of *Linum usitatissimum* [8], as well as a paper on *Nicotiana* nectar chemical profiles [9]. The article by Pontarin et al. [8] is an excellent example showing the complexity of plant metabolome, several concentration kinetics along time were presented, covering several compound classes.

Some papers made an in-depth analysis of both specialized and primary metabolites. These include a study on the variability of rapeseed compounds [18], a paper on metabolite changes in the important medicinal plant *Pelargonium sidoides* as a response to irrigation and nitrogen [19], as well as an excellent study on metabolomic response in grapevine wood after infection with a fungal pathogen, *Neofusicoccum parvum* [6].

The field is not without challenges; it is a rapidly evolving one with many issues upfront which need to be resolved in the future. First, while we have widely accepted protocols for quality control of the measurements that quantify compounds after calibration with authentic standards, the quality control in metabolomics is still a developing field [20]. As there are drifts in the sensitivity of the instruments, one has to execute special measures such as randomized injection order, application of quality control (QC) samples, LOESS fitting, among others [21], otherwise, merging datasets measured years after one another in large cohort studies would be rendered impossible. A series of QC (quality control) samples are usually used to resolve this issue, though there is a debate about how an optimal QC sample set is to be prepared, and all solutions have their compromises [22].

Compound annotation and data interpretation of data is also not an easy task—while transcriptomics and proteomics yield sequences that can be subjected to at least similarity studies, the identification of small molecules is not that straightforward as the structural variability is much higher [23]. This step is likely the most critical bottleneck of the metabolomic workflow recently. Putative annotation uses database matches, and many times results, in compounds that are likely the result of over-fitting—results can be like erroneously “finding” a wide array of halogenated pharmaceuticals in field plants because of improper database matches. Several tools exist that offer partially or fully automated annotation or extraction of chemical information from the MS/MS spectra [24,25,26,27], but results are always to be handled with special care. As a consequence, most features found in a study remain unannotated [23]. Data pre-treatment and evaluation is not less sensitive [28].

I think we all look forward to the upcoming advancements of this excellent technique in the future. Hereby, I would like to thank the authors for their contribution, the peer reviewers who helped scientific evaluation of the submissions, as well as the staff members of the Metabolites Editorial Office.
